# Supplementary material for: Oral Administration of Lipopolysaccharide Enhances Insulin Signaling-Related Factors in the KK/Ay Mouse Model of Type 2 Diabetes Mellitus
Source: Int J Mol Sci. 2023 Feb 27;24(5):4619. doi: 10.3390/ijms24054619 (PMC10003108; doi:10.3390/ijms24054619)
Supplement: Supplementary file 1 [file ijms-24-04619-s001.zip › ijms-2220300-supplementary.pdf]

Oral Administration of Lipopolysaccharide Enhances Insulin Signaling-Related Factors in the KK/Ay Mouse Model of Type 2 Diabetes Mellitus

Supplementary Figure S1

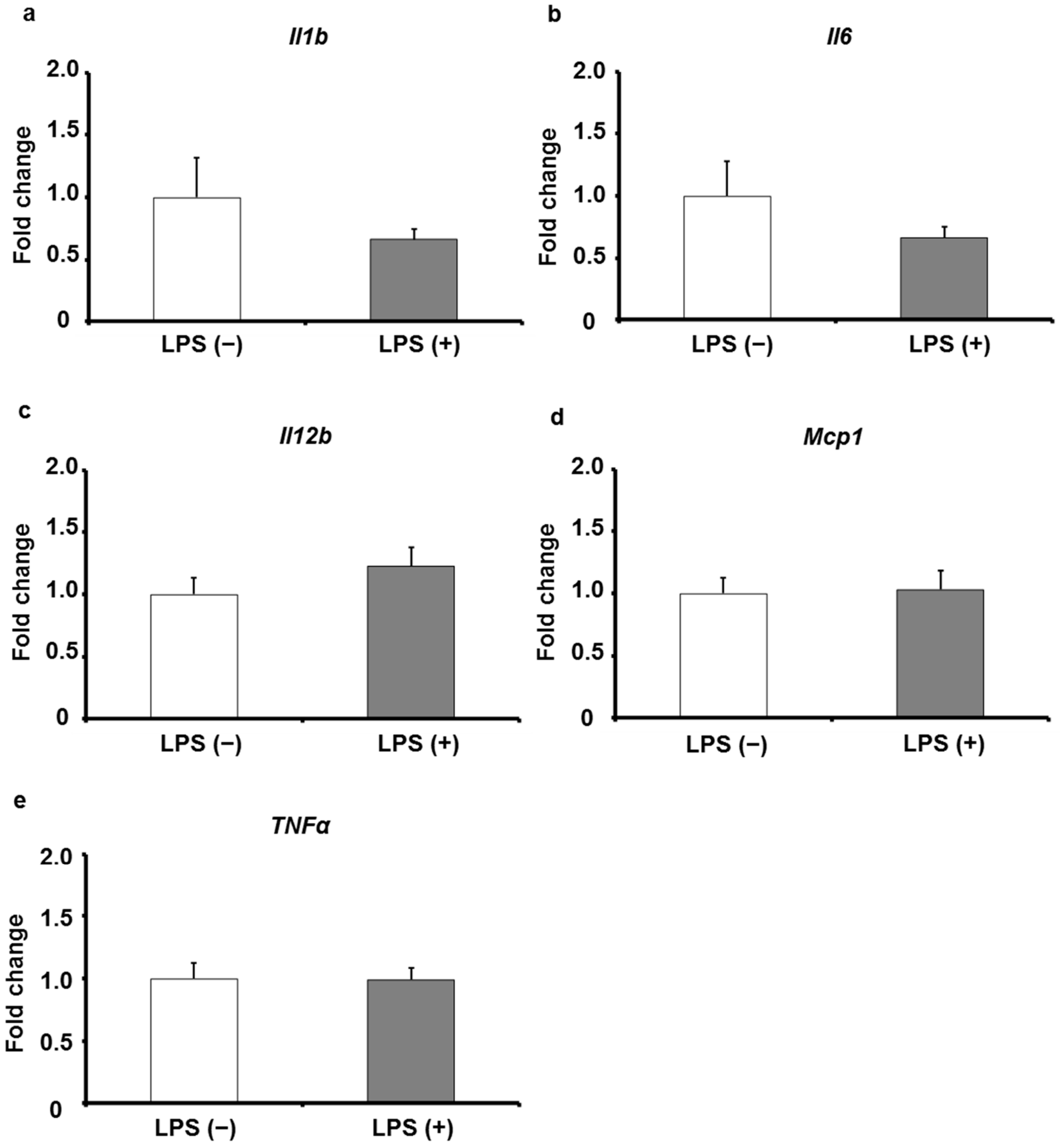

Supplementary Figure S1. Effects of orally administered LPS on cytokine expression in adipose tissues of KK/Ay mice. The relative mRNA expression levels of (a-e) Interleukin 1 beta (*Il1b*), Interleukin 6 (*Il6*), Interleukin 12 beta (*Il12b*), Monocyte chemoattractant protein-1 (*Mcp1*), and Tumor necrosis factor-*α* (*TNFα*) were measured by qRT-PCR ($n = 8$). Values are presented as the mean ± SE. *p <0.05 vs. LPS (−) group.

Oral Administration of Lipopolysaccharide Enhances Insulin Signaling-Related Factors in the KK/Ay Mouse Model of Type 2 Diabetes Mellitus

Supplementary Figure S2

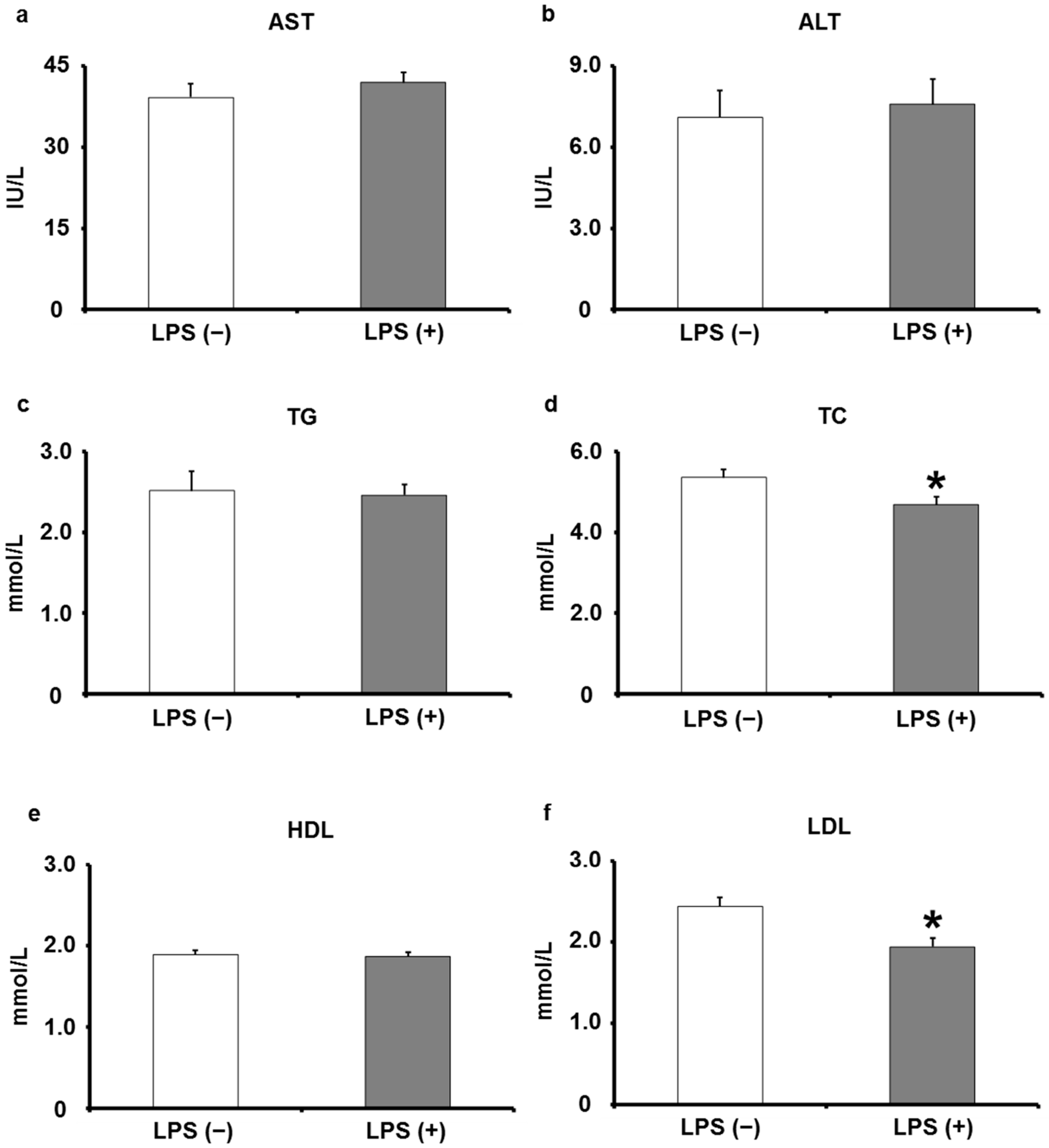

Supplementary Figure S2. Effects of orally administered LPS on biochemical parameters in KK/Ay mice. (a-f) Aspartate transferase (AST), alanine transaminase (ALT), triglycerides (TG), total cholesterols (TC), high-density lipoprotein (HDL), and low-density lipoprotein (LDL) levels were measured using commercial kits (n = 8). Values are presented as the mean ± SE. *p <0.05 vs. LPS (−) mice.
